# Poster Session II - A278 DISTINCT CLINICAL AND PATHOLOGIC CHARACTERISTCIS OF UC-ASSOCIATED NEOPLASIA AMONG PATIENTS UNDERGOING COLECTOMY: A 15-YEAR SINGLE-CENTRE COHORT STUDY

**DOI:** 10.1093/jcag/gwaf042.278

**Published:** 2026-02-13

**Authors:** J Kim, I Hebert-milette, J Conner, K Borowski, J Stempak, S Lee, M Silverberg

**Affiliations:** Zane Cohen Centre for Digestive Diseases, Mount Sinai Hospital, Toronto, ON, Canada; Zane Cohen Centre for Digestive Diseases, Mount Sinai Hospital, Toronto, ON, Canada; Zane Cohen Centre for Digestive Diseases, Mount Sinai Hospital, Toronto, ON, Canada; Zane Cohen Centre for Digestive Diseases, Mount Sinai Hospital, Toronto, ON, Canada; Zane Cohen Centre for Digestive Diseases, Mount Sinai Hospital, Toronto, ON, Canada; Zane Cohen Centre for Digestive Diseases, Mount Sinai Hospital, Toronto, ON, Canada; Zane Cohen Centre for Digestive Diseases, Mount Sinai Hospital, Toronto, ON, Canada

## Abstract

**Background:**

Despite advancements in endoscopic surveillance approaches and medical therapies, ulcerative colitis (UC)-associated neoplasia remains a major concern in patient care and often necessitates colectomy.

**Aims:**

This study aimed to compare the clinical characteristics of patients with UC-associated neoplasia with those of medically refractory UC who underwent colectomy, and to further characterize the pathological features of UC-associated neoplasia.

**Methods:**

We retrospectively reviewed medical records of patients with UC who underwent colectomy between January 2010 to July 2024. Patients were categorized as having medically refractory disease (MR) or UC-associated neoplasia as their indication for colectomy. Clinicopathological characteristics were compared between groups, and multivariate logistic regression was performed to identify factors associated with colectomy indications.

**Results:**

A total of 486 patients were eligible for analysis, comprising 356 (73.6%) with MR and 130 (26.7%) with UC-associated neoplasia. Patients in the neoplasia group were older at colectomy (median 47 vs 34 years, p < 0.001) and had a longer disease duration (16 vs 4 years, p < 0.001). In multivariate analysis, concurrent steroid use was ten times more likely in the MR group (Odds ratio [OR] 10.1, 95% confidence interval [CI] 3.9-28.8, p < 0.001), while PSC was ten times more likely in the neoplasia group (OR 0.1, 95% CI 0.05-0.20, p < 0.001). In the neoplasia group, 44.8% had neoplasia in 2 or more colonic segments and 67.7% showed advanced lesions including high-grade dysplasia or adenocarcinoma. When comparing the most advanced findings in preoperative biopsies compared with surgical pathology, 31.9% (37/116) showed discordant findings. Among these, 42.1% (16/38) with low-grade dysplasia and 25.7% (9/35) with high-grade dysplasia on biopsy were found to have more advanced lesions in the colectomy specimens.

**Conclusions:**

Significantly longer disease duration and a high rate of PSC were observed in patents with UC-associated neoplasia. Most neoplasia cases exhibited histologically active inflammation in surgical specimens, and a substantial portion showed multifocal or advanced lesions, Notably, over one-third of patients with dysplasia on biopsy had more advanced pathology at colectomy. These findings underscore the importance of meticulous endoscopic and histologic surveillance in long-standing UC even in presence of active inflammation.

**Funding Agencies:**

NIH, NIDDK, Hold’em for Life Charity Challenge

